# Femoral Hernia Containing a Strangulated Appendix: A Hybrid Approach

**DOI:** 10.7759/cureus.37202

**Published:** 2023-04-06

**Authors:** Paraskevi Dedopoulou, Athanasios Papatriantafyllou, Konstantina Soukouli, Ioannis Karioris, Stylianos Tsochatzis

**Affiliations:** 1 Department of Surgery, General Hospital of Patras, Patras, GRC

**Keywords:** surgical treatments of hernias, atypical appendicitis, incarcerated femoral hernia, femoral hernia, de garengeot hernia

## Abstract

We present an intriguing case of an incarcerated femoral hernia containing an inflamed appendix inside the sac, also known as a De Garengeot hernia. This type of hernia is a rare occurrence that was first described in 1731 by the French surgeon René-Jacque Croissant de Garengeot. A 64-year-old woman presented to the emergency department with a painful mass in the right groin region. Following a computed tomography (CT) scan of the abdomen and pelvis to evaluate the mass, the diagnosis of a femoral hernia containing a strangulated appendix was established. Subsequently, a hybrid surgical approach was utilized, consisting of an open hernia repair and a laparoscopic appendectomy.

## Introduction

A hernia is defined as the protrusion of any intraperitoneal organ or part of it through a weak area or hole in the abdominal wall. The classification of a hernia is based on its location and the content of the hernia sac. The most commonly occurring types of hernias include inguinal hernias, femoral hernias, incisional hernias, umbilical hernias, and epigastric hernias [1]. Inguinal hernias occur through a weak spot located above the inguinal ligament, while femoral hernias occur through a weak spot located below the inguinal ligament. The most common contents of groin hernias are the omentum and loops of the small intestine. However, uncommon contents of the hernia sac may include the appendix, fallopian tube, or ovary in women, the urinary bladder, Meckel’s diverticulum, and the sigmoid colon [1, 2]. When the vermiform appendix is the content of the hernia sac in an inguinal hernia, it is known as an "Amyand's hernia." In contrast, when the appendix is present in a femoral hernia sac, it is referred to as a "De Garengeot hernia", as was the case in our patient. The De Garengeot hernia is a relatively uncommon type of femoral hernia, accounting for 0.5%-5.0% of all femoral hernias. The percentage of appendicitis inside the femoral hernia is even lower, ranging from 0.08%-0.13% [3, 4]. It is more prevalent in women [5]. Due to its rarity, diagnosing this type of hernia can be particularly challenging. In most cases, the diagnosis is made intraoperatively, and prompt surgical intervention is recommended to prevent potential complications such as appendix perforation and peritonitis.

## Case presentation

**Figure 1:**
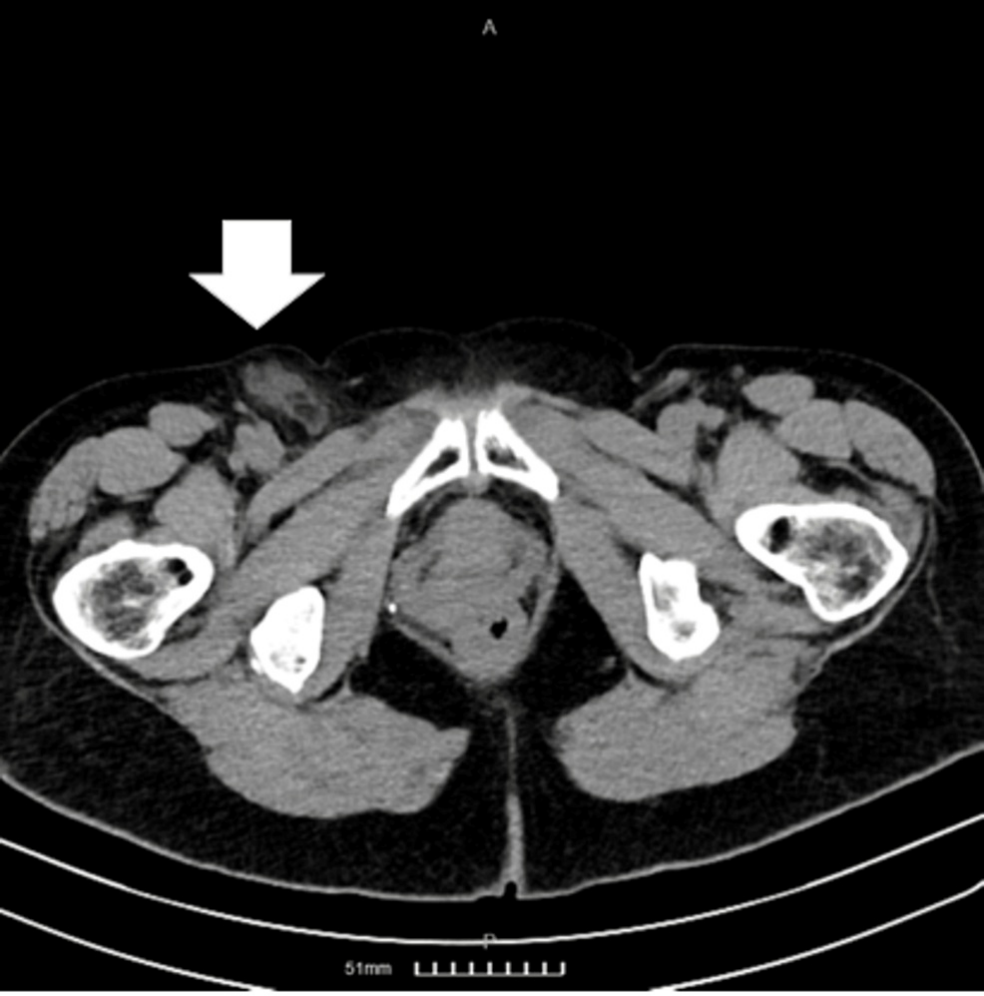
An axial view of the CT showing a femoral hernia containing the appendix

We report the case of a 64-year-old woman who presented to the emergency room with sudden-onset pain in her right groin following a weightlifting incident. She did not report any symptoms of bowel obstruction or any significant medical history. Physical examination revealed a tender, non-mobile, and painful mass in the right groin. Blood tests showed, respectively, normal levels of C-reactive protein (0.1 mg/dL) and white blood cell count (7.8K/μL). A CT scan of the abdomen and pelvis was performed, revealing a right femoral hernia containing a strangulated appendix (Figure 1).

**Figure 2:**
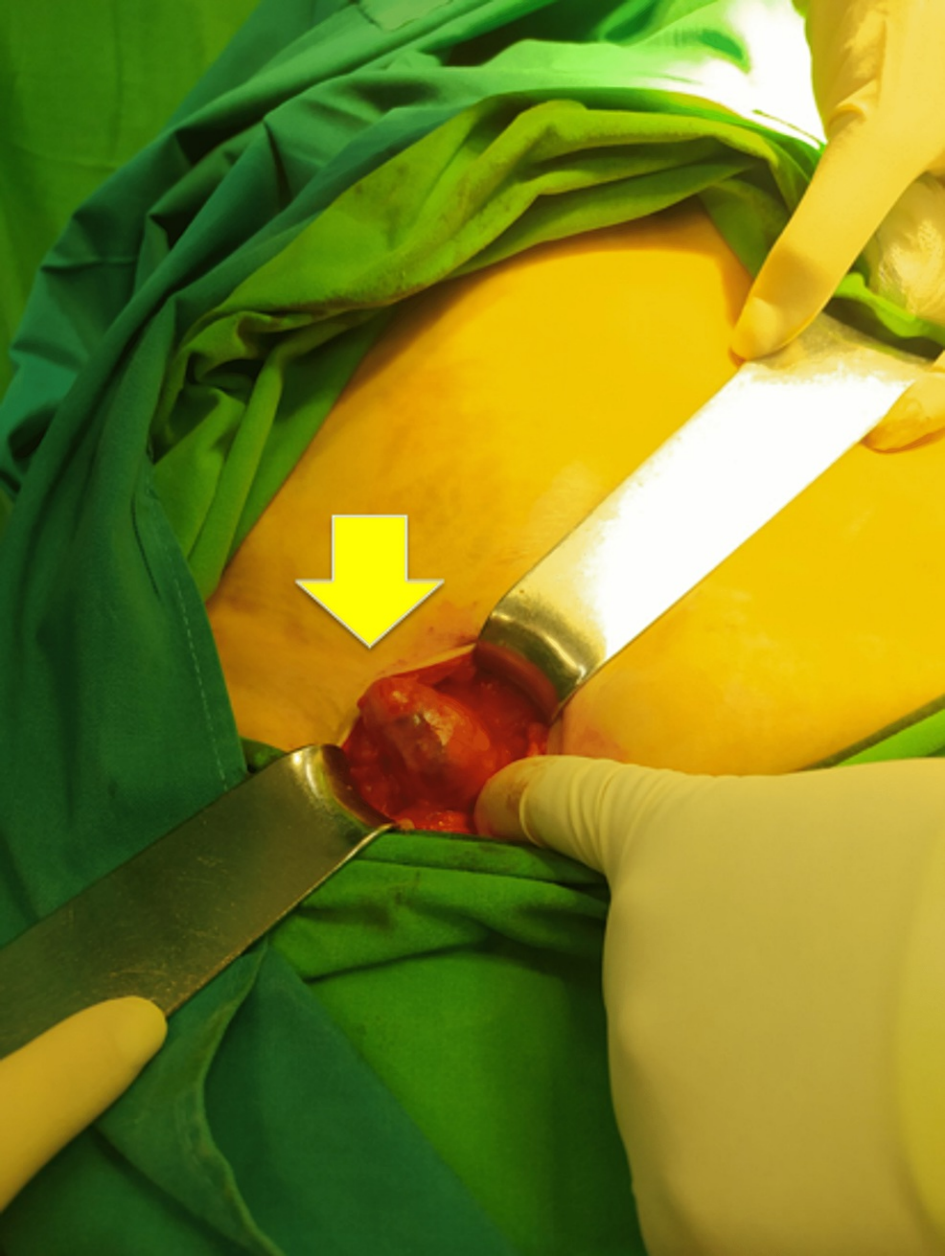
A photo from the operation shows a femoral hernia containing the appendix

The patient underwent surgical intervention, beginning with an open repair of the hernia. Upon opening the sac, an inflamed appendix was observed and reduced back into the peritoneal cavity (Figure 2).

The hernia was repaired using sutures. The wound was closed with sutures. We then proceed to perform a laparoscopic appendectomy. The patient had an uneventful postoperative recovery and was discharged two days after the surgery.

## Discussion

The diagnosis of this hernia subtype may pose a challenge; however, a computed tomography (CT) scan of the abdomen and pelvis can be a valuable diagnostic tool to aid in the preoperative diagnosis of a De Garengeot hernia [6].

An immediate and accurate diagnosis is crucial in determining the appropriate surgical plan. A majority of femoral hernias present as an emergency condition due to the narrow neck of the femoral ring. Due to the rarity of this type of hernia, no specific surgical technique is used. In most cases, the inguinal approach is preferred, which allows for both hernia repair and appendectomy. In cases where laparotomy is selected, it is due to the quality or accessibility of the base of the appendix and the need for exploration of the abdominal cavity. For cases requiring laparotomies, a McBurney incision or midline incision is typically chosen for better access [5].

In the inguinal approach, the McEvedy technique (preperitoneal) appears to lead to fewer laparotomies compared to the Lockwood (infrainguinal) and Lotheissen's approaches (transinguinal). In cases like ours, there is a choice to combine laparoscopic appendectomy and open hernia repair. Laparoscopy provides the option to explore the abdomen and perform a laparoscopic appendectomy. In some cases, the laparoscopic approach was used for both appendectomy and hernia repair. Transabdominal preperitoneal repair (TAPP) and total extraperitoneal repair (TEP) have also been used in combination with laparoscopic appendectomy in the case of Garengereot hernias [5].

Regarding hernia repair, sutures were predominantly used. However, in selected cases where there were no indications of severe inflammation of the appendix, a mesh was employed.

In this case, the patient underwent a successful open hernia repair and laparoscopic appendectomy. By means of laparoscopy, we had the opportunity to assess the abdominal cavity and perform a laparoscopic appendectomy. The preoperative diagnosis of a De Garengeot hernia was essential in selecting the appropriate surgical approach for our patient.

## Conclusions

This case underscores the significance of an expeditious and comprehensive assessment of a femoral hernia, as it may harbor diverse contents that have the potential to result in grave complications. The effective management of our patient's De Garengeot hernia necessitated a prompt and precise diagnosis, followed by appropriate surgical intervention. Therefore, it is imperative to incorporate the possibility of a De Garengeot hernia in the differential diagnosis of all patients who present with a groin mass.
